# A long-term analysis, modeling and drivers of forest recovery in Central Mexico

**DOI:** 10.1007/s10661-024-13584-0

**Published:** 2024-12-21

**Authors:** José López-García, Gustavo Manuel Cruz-Bello, Lilia de Lourdes Manzo-Delgado

**Affiliations:** 1 Instituto de Geografía, Universidad Nacional Autónoma de México, Ciudad Universitaria, C.P. 04510 Coyoacán, Mexico City, Mexico; 2 Departamento de Ciencias Sociales, Universidad Autónoma Metropolitana, Unidad Cuajimalpa. Avenida Vasco de Quiroga 4871, Col. Santa Fe, C.P. 05348 Mexico City, Mexico

**Keywords:** Land cover change, Reforestation, Natural regeneration, Socioeconomic drivers, Predictive modeling, Conservation policies

## Abstract

This study aims to evaluate the changes in forest cover from 1994 to 2015, identify the key drivers of forest recovery, and predict future trends. Using high-resolution remote sensing data, we mapped forest canopy density into detailed categories (closed > 50%, open 10–50%, and deforested < 10%) to differentiate processes like degradation, deforestation, densification, reforestation, and afforestation. A multinomial logistic regression was used to explore the relationship between the forest processes and socioeconomic, proximity, planning, and policy potential drivers. Future trends were modeled using the Land Change Modeler. The analysis showed that 81.5% of the area remained unchanged, 14% experienced recovery, and 4.5% faced disturbances. Factors such as elevation, proximity to roads, and participation in payment for environmental services (PES) programs significantly influenced recovery trends. Predictive modeling for 2035 suggests forest cover will increase by 7%, reaching 77% coverage of the study area, and closed forest areas will rise by 12% compared to 1994. The findings underscore the effectiveness of conservation efforts and natural regeneration in enhancing forest cover, offering valuable insights for global forest management and policy-making efforts.

**Supplementary Information:**

The online version contains supplementary material available at 10.1007/s10661-024-13584-0.

## Introduction

Globally, 178 million hectares of forest were lost between 1990 and 2020, even though the net forest loss rate slowed due to reduced deforestation in some countries and an increase in forest area through afforestation and natural expansion of forests in others (FAO, 2020a). Although deforestation has dominated land use change, the socioeconomic changes and the abandonment of agricultural land and pasture have increased the secondary forest cover in many regions (Li & Li, 2017; FAO & UNCCD, 2015; Song et al., 2018). The regeneration of natural forests reflects a decline in traditional agricultural practices that can be observed worldwide (Gellrich et al., 2007). Natural forest regeneration on former agricultural lands can contribute to biodiversity conservation, climate change mitigation and adaptation, and the provision of multiple ecosystem services (Houghton et al., 2015; Pugh et al., 2019; Wilson et al., 2017), as well as to improve food security and protect water supplies in a socially, economically, and ecologically effective way (Chazdon & Brancalion, 2019). Despite social and ecological pressures, forests are regenerating in many world regions (Hecht et al., 2014). Similar benefits can be provided through active forest restoration, but at significantly higher costs (Bullock et al., 2011). Natural regeneration (passive reforestation) may not overcome forest loss, requiring active restoration through site preparation and tree planting (Holl & Aide, 2011; Holl et al., 2018).

Dependence on forests is underpinned by international initiatives to halt deforestation and increase the restoration of degraded forests worldwide, such as the 2011 Bonn Challenge. The Aichi target is to restore at least 15% of degraded ecosystems by 2020 (CBD, 2011; Verdone & Seidl, 2017). This has contributed to an increase in tree cover of 2.24 million km^2^ (up 7.1% from the 1982 level) (FAO, 2020a). Global-scale analysis of satellite imagery from 1982 to 2016 revealed that tree cover is changing dramatically in major geographic regions, with tree cover gain attributed to natural regeneration and tree plantation establishment (Song et al., 2018). An analysis of 166 studies conducted on forests that were naturally regenerating or actively restored worldwide demonstrated that forests exhibit a remarkable capability for recovery (Meli et al., 2017). Studies have found disparities in forest recovery between developed and developing countries. Benedek and Fertő (2020) observed that middle-income nations have experienced the smallest gains in the quality and quantity of new forests. And that economic development can lead to a long-term decline in deforestation and an increase in the extent and quality of forests. However, this process usually begins with a rapid recovery period followed by a prolonged slowdown in forest recovery. The trend eventually turns positive after reaching a low point, but only the richest countries have reached this transition point.

Deforestation and forest degradation are still major issues in some developing countries (Mehmood et al., 2024; Song et al., 2018), where there is often a lack of quantitative data on past trends (Suratman & Latif, 2020). However, in developed regions, there is significant afforestation and forest regrowth, partly due to the implementation of land use change, urbanization, and reforestation policies (Hecht et al., 2016). Net increases in tree cover detected from satellite imagery in boreal and temperate biomes between 2000 and 2010 can be primarily explained by natural forest regeneration on abandoned agricultural land (FAO & UNCCD, 2015). Latin America and the Caribbean experienced extensive deforestation and reforestation between 2001 and 2010 (Aide et al., 2012). This study estimated a net forest loss of 179,405 km^2^, resulting from 541,835 km^2^ deforested and 362,430 km^2^ reforested. Between 2001 and 2014, five general types of neotropical reforestation hotspots were defined, reflecting topographic features and aspects related to agroecological marginality, climate change, rural population decline, and increased urbanization (Nanni et al., 2019).

Reforestation intensified in Central Mexico, in 2003 with the start of the federal government's payment for environmental services (PES) (PROBOSQUE, 2016) and in 2007 with the implementation of the State of Mexico PES program (CONAFOR, 2007) for the restoration of the Cutzamala basin system, on which 37% of the water consumed in Mexico City depends (CONAGUA, 2005). Honey-Rosés et al. (2018) found that from 1986 to 2012, most of the recovered forests in a large forest area surrounding Monarch Butterfly Biosphere Reserve (MBBR) resulted from passive recovery due to the abandonment of the agricultural areas, attributable to the low profitability, the youth emigration, and the farm intensification. Assisted regeneration of agricultural lands through afforestation and active and passive reforestation of deforested lands is responsible for recovering forests in Central Mexico (López-García & Navarro-Cerrillo, 2021). Forest recovery in one region of Michoacán seems to be more influenced by local and regional factors than by government programs (Špirić et al., 2023), so it is more likely that it occurred passively through regeneration and densification following fires, agricultural abandonment, or reduction of illegal logging and grazing (Honey-Rosés et al., 2018; Manzo-Delgado et al., 2014).

Central Mexico presents a unique blend of ecological, economic, and governance factors influencing land use and land cover changes. It encompasses diverse mountainous landscapes with varied elevations and climates, making it a biodiversity hotspot (Rico-Sánchez et al., 2020). These geographic features, ongoing human activities, such as agriculture, and proximity to large urban centers, such as Mexico City, create a dynamic environment. In addition, it contains numerous protected areas and communal lands where conservation efforts, reforestation programs, and payments for environmental services are actively pursued (López-García & Navarro-Cerrillo, 2021). This makes the region a critical case study for examining how governance and socioeconomic changes impact forest recovery and degradation.

Canopy cover measurements are essential to accurately reveal the progress of forest recovery or degradation beyond simply estimating forested areas (Tang et al., 2019). Measuring forest canopy density, which indicates the proportion of the ground covered by tree crown cover, is crucial for understanding forest health, structure, biomass, and habitat quality (Bhandari, Nandy, 2024; Xu et al., 2024). Canopy density influences light availability within the forest, affecting plant growth, species interactions, and ecosystem processes (Parker et al., 2019). High canopy density is often characteristic of mature, undisturbed forests, which play a vital role in biodiversity conservation and climate regulation. In contrast, lower canopy density can indicate forest degradation, deforestation, or transitional ecosystems like young regrowing forests (Hiroaki et al., 2004).

In this context, this study aims to evaluate the changes in forest cover over 21 years, identify the key drivers of forest recovery, and predict future trends. The research advances the methodology for forest cover mapping by classifying forest canopy density into fine categories (closed, open, deforested, and non-forest) and allowing the differentiation of specific forest dynamic processes (degradation, deforestation, densification, reforestation, and afforestation). This detailed classification enhances the accuracy of forest monitoring, and the predictive model forecasts future forest cover trends, providing a basis for long-term planning and conservation strategies.

## Methods

### Study area

**Fig. 1:**
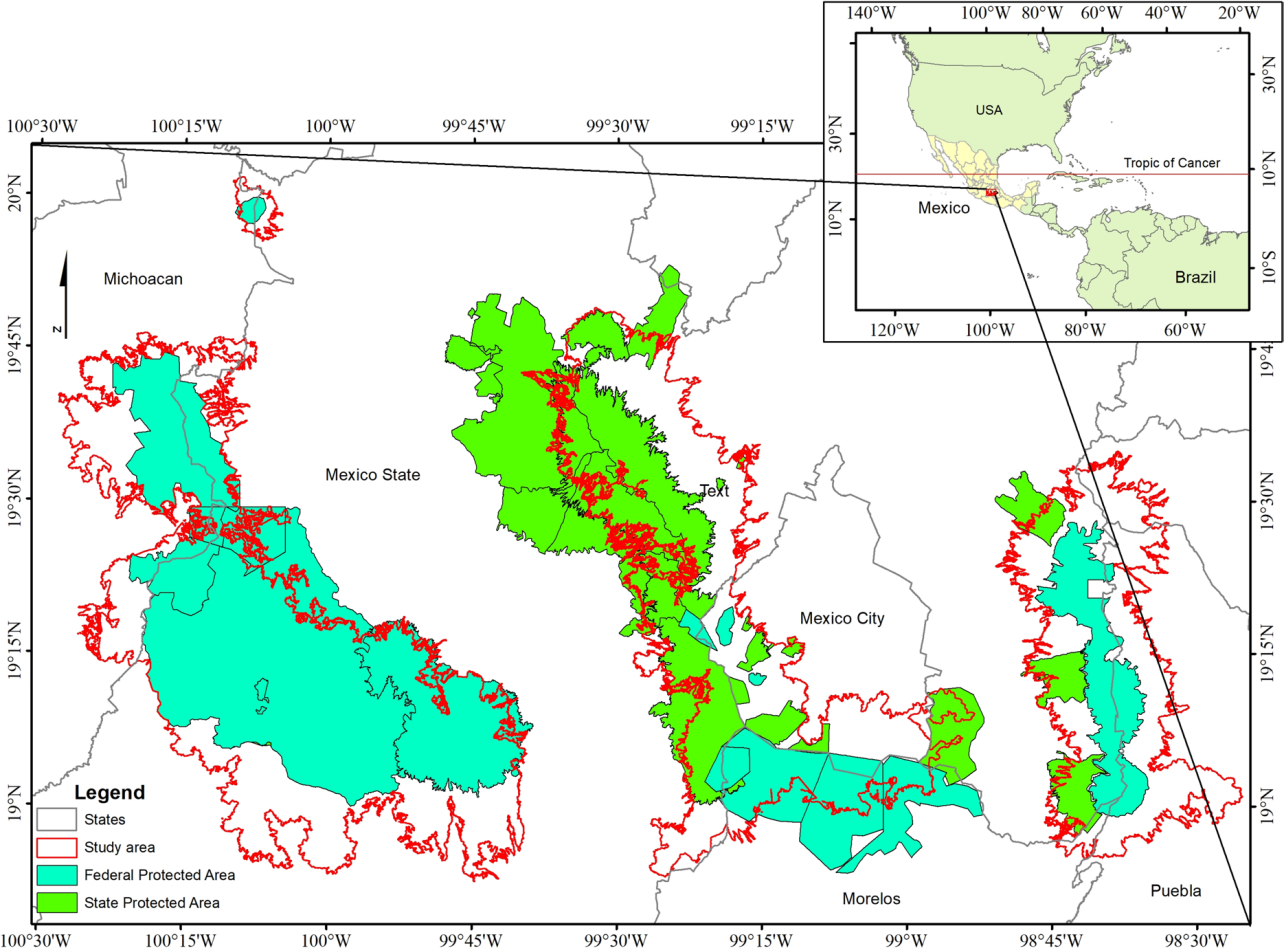
Study area, including protected areas and state boundaries

The study area covers 735,282 ha, ranging from 1,300 to 5,400 m a.s.l. It is in central Mexico and includes four mountainous zones of temperate forests: Angangueo-Valle de Bravo-Nevado de Toluca, Sierras de las Cruces-Ajusco-Chichinautzín, Sierra Nevada-Volcán Tlaloc, and Cerro Altamirano. Temperate forests represent the most important forests in the region. They are important for hydrological and forest resources, whose species include *Abies religiosa* at altitudes of 2900–3600 m, *Pinus hartwegii*, *P. montezumae*, *P. ayacahuite*, *P. teocote*, *P. pseudostrobus* between 2400 and 2900 m altitude, and some associated species of the genera *Quercus*, *Arbutus* and *Alnus *below 2400 m altitude. It hosts all or part of thirty protected areas: thirteen federal (seven national parks, two flora and fauna protection areas, two conservation areas, one biosphere reserve, and one natural resource protection area) and fifteen state (eleven state parks, three community ecological areas, and one nature reserve) (Fig. 1). The area comprises 113 municipalities distributed in the states of Mexico, Morelos, Puebla, Michoacán, and Mexico City. The lowlands surrounding the mountainous regions are home to cities with a high concentration of inhabitants: Mexico City Metropolitan Zone (twenty million inhabitants), Toluca (418,569 inhabitants), and Cuernavaca (378,476 inhabitants) (INEGI, 2020).

### Data

To determine the land cover in the study area, we used 198 panchromatic orthophotographs from 1994 at 2 m per pixel (February–March 1994) obtained from the INEGI (the Mexican government's geographical and statistics agency) and five SPOT-6 images at 1.5 m per pixel (February 2015) from the receiving station México-Nueva Generación (ERMEX-NG) (SIAP, 2017). We generated a mosaic for the orthophotos and another for the SPOT satellite images. All layers were re-projected to Universal Transverse Mercator (UTM) 14N, WGS84.

### Interpretation of forest canopy density

Considering the continuous forest stands delimited in the 1994 orthophotographs, the study area was defined and regarded as the study baseline. The density of the forest cover was digitized by visual interpretation of the orthophotos at a scale of 1:5,000 on a PC screen, using ArcGis 10.8. The elements of image photo interpretation were considered, such as tone, texture, shape, design, and relationship with other objects (Horning, 2004). This study assessed changes in forest cover density over 21 years in temperate forests of central Mexico, examining the factors driving forest recovery. We used high-resolution orthophotographs and SPOT-6 imagery to accurately map three forest canopy densities by visual interpretation of the different forest cover densities (qualitative estimation), verified in the field by circular samples of 1000 m^2^ to establish the average number of trees per hectare, which allowed to separate the different forest densities: closed > 50% and open 10–50% (López-García et al., 2016) and deforested < 10% (FAO, 2010), and identify the processes of degradation, deforestation, densification, reforestation, and afforestation (See the Supplementary Information for three densities examples). Once the 1994 cover density map was completed, the topology was checked, and the layer errors were corrected. Using the interdependent classification (FAO, 2020b), the 1994 forest cover density map provided the basis for the 2015 cover density map, so only the polygons that changed were modified. The 2015 map was edited and overlaid on the SPOT-6 image mosaic to edit for changes in forest cover density, the topology was revised, and errors were corrected.

The 1994 and 2015 cover maps were combined to identify changes within and outside protected areas over this period. This allowed us to determine six change processes defined by FAO (2010) over the 21 years. Three were negative changes, Forest degradation (decrease in forest cover density), Deforestation (reduction in cover density to < 10%), and Land use change (hereafter referred to as transformation from forest to another use). Three were recovery processes, Densification (increase in forest cover density), Reforestation (Restoration of the forest through planting or deliberate seeding on land classified as forest), and Afforestation (Establishment of forest by deliberate planting or seeding on land that has not until then, been classified as forest), since there was no evidence to the contrary, it was assumed that any land that in 1994 did not have forest was land that, until then, was not classified as forest. Persistence refers to areas that did not show changes in forest density.

### Analysis of forest cover change

The resulting 1994 and 2015 maps were combined to create a change matrix (Table 1). Values in the shaded diagonal represent persistence. The transitions below the diagonal correspond to recovery processes, and those above are considered disturbance processes. These processes were divided according to the degree of recovery/disturbance occurring over these 21 years (Table 1).

**Table 1:**
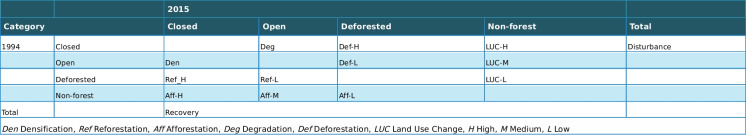
Forest cover change matrix and the resulting processes. Recovery processes below the diagonal and disturbance processes above the diagonal

*Den* Densification, *Ref* Reforestation, *Aff* Afforestation, *Deg* Degradation, *Def* Deforestation, *LUC* Land Use Change, *H* High, *M* Medium, *L* Low

The matrix allowed the differentiation of various processes within forest dynamics. The processes of change were defined according to FAO (2000). The net change in the forest area during a specific period was derived from the sum of all changes: disturbances due to degradation, deforestation, or land use change and recovery due to forest densification, reforestation, or afforestation (FAO, 2020a). We also determined which categories converted to other categories between 1994 to 2015.

**Fig. 2:**
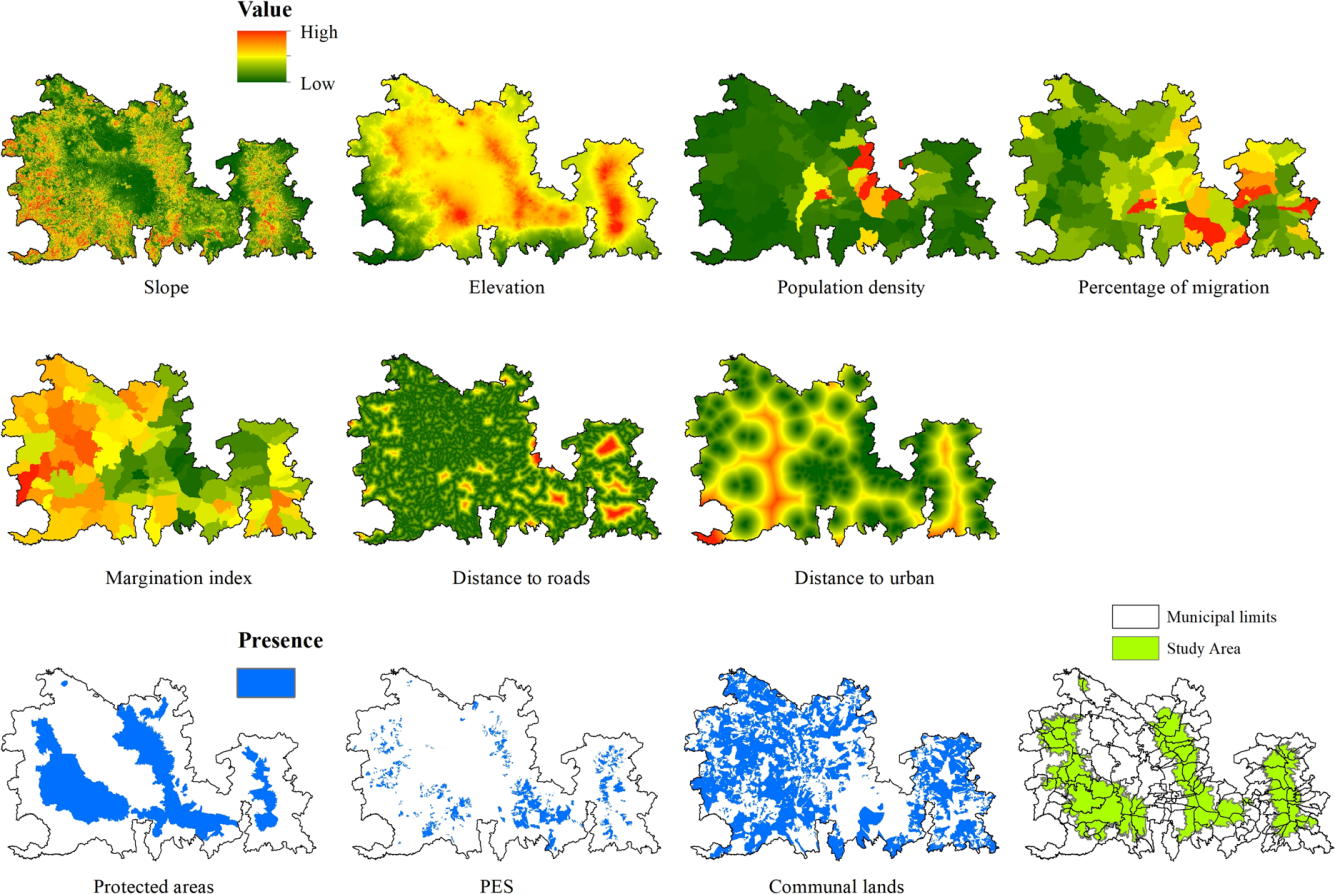
Potential forest recovery drivers

We explored the relationship between the forest processes Recovery, Persistence, and Disturbance and some of the socioeconomic, proximity, site factors, and planning and policy variables reported as significant drives of land use and land cover change (Camacho, 2022; Naikoo et al., 2023). Specifically, we used slope and elevation (INEGI, 2013) for the physical type, population density (INEGI, 1995a), average percentage of migration (SEGOB, 2022), and the margination index (deficiencies suffered by the population due to the lack of access to education, inadequate housing, and the lack of property; CONAPO, 2020) for the socioeconomic kind. For the proximity dimension, we used distance to roads (INEGI, 1995b) and distance to urban (INEGI, 1997); for the planning and policy type, we used protected areas (CONANP, 2023), areas with payment for environmental services (CONAFOR, 2011), and areas of communal lands (ejidos and communities) (RAN, 2013). The limits of the municipalities from which the socioeconomic drivers were derived were used to delimit all other drivers (Fig. 2).

For our exploratory analysis, we used a multinomial logistic regression in SPSS (IBM Corp., 2011). This regression analysis is used when the dependent variable is nominal and has more than two categories. It can be used with any number of independent categorical or continuous variables (Long & Fresse, 2014). This model estimates the logarithmic probabilities (logits) of an area falling into a category of the dependent variable other than the reference category (Hosmer et al., 2013), which in this case was Persistence. The protected areas, the areas with payment for environmental services, and the areas of communal lands were included as binary predictors.

Additionally, a Chi-square test was performed to specifically determine the relationship between PES areas and those in which a recovery process occurred.

### Modeling of forest cover change

Finally, we modeled the future land use change through the Land Change Modeler (LCM) available in TerrSet (Clark Labs, 2024). The LCM is an empirical tool to analyze land use and land cover changes, modeling future scenarios, and assessing environmental impacts. It calculates land use change using a transition probability matrix constructed through Markov chain analysis and a transition potential layer based on two historical land cover layers. It produces susceptibility maps that rank the change potential using logistic regression or machine learning techniques, including multi-layer perceptron neural networks (Eastman & Toledano, 2018). Its key strengths are its dynamic projection proficiency, appropriate calibration, and ability to simulate diverse land cover types (Leta, et al., 2021). Still, its performance is limited by data quality and predefined algorithms that may not capture complex socio-environmental dynamics.

We used Cramer´s V statistic to select the model's input variables to predict the future land use in the study area. The Cramer's V statistic is a correlation measure between categorical variables ranging from 0 to 1, where 0 indicates no association and a value of 1 indicates a perfect relationship. The specialized literature in LUCC considers a variable to be associated with a particular type of land use/cover if the value of this statistic is near 0.15 or greater (Shooshtari & Gholamalifard, 2015; Subiyanto & Suprayogi, 2019). The transition potential between categories was computed through the multi-layer perceptron (MLP) neural network model. The transition probability matrix was calculated using Markov Chain computation. Since LCM lacks a mechanism to restrict forest expansion into high-elevation areas where the only land cover is grass or snow, all forest expansion above 4,000 m a.s.l. was kept in the No-forest category present in 2015. This altitudinal threshold was taken from Rzedowski (2006) and Soto et al. (2021), who reported it as the upper limit of forest cover in central Mexico.

## Results

Temperate forests recovered between 1994 and 2015 in the central region of Mexico, highlighting that 81.5% of the study area remained unchanged, while 18.5% had some losses or gains. Of the change, 14% corresponded to recovery, and 4.5% were disturbances. In 1994, the forests covered an area of 513,958 ha (69.9%). They increased by 33,043 ha, an increase of 4.5% of the total area in 21 years, indicating forest recovery at the expense of deforested and non-forest areas dedicated to agriculture or livestock. Specifically, closed forests dominated and increased by 58,987 ha (8%) (Table 2).

**Table 2:** Forest cover change 1994–2015

		2015				
	Category	Closed	Open	Deforested	Non-forest	Total
				(ha)		
1994	Closed	405,549	12,075	6,150	4,305	428,079
	Open	50,213	26,895	5,197	3,570	85,875
	Deforested	9,217	11,785	32,498	2,051	55,551
	Non-forest	22,087	9,176	0	134,512	165,775
	Total	487,066	59,931	43,845	144,438	735,280

Densification was the primary recovery process, followed by afforestation and reforestation. Forest degradation represented 1.6% of the disturbance processes, followed by deforestation and land use change (Table 3). In the final balance, densification dominated forest degradation, reforestation exceeded deforestation, and afforestation dominated land use change (Table 3).

**Table 3:** Land cover change processes

Process	ha	% Absolute	% Relative Process Group
Degradation	12,073	1.6	36.2
Deforestation	11,348	1.5	34.0
LUC	9,928	1.4	29.8
Disturbance	33,349	4.5	100
Persistence	599,445	81.5	100
Densification	50,221	6.8	49.0
Reforestation	21,002	2.9	20.5
Afforestation	31,256	4.3	30.5
Recovery	102,480	14.0	100
Total	735,273	100	

**Fig. 3:**
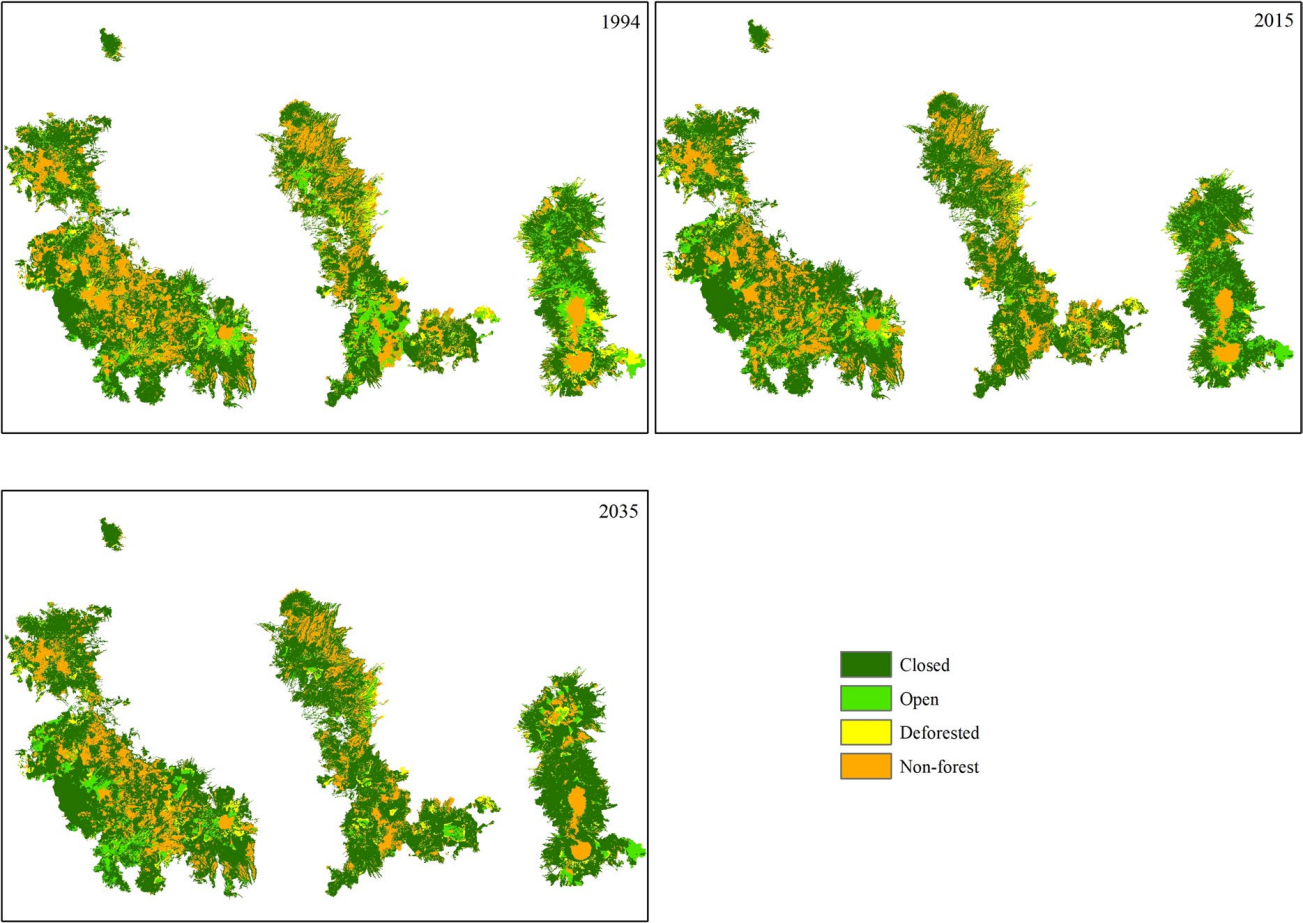
Forest cover density for 1994, 2015, and prediction to 2035

The cover density matrix between 1994 and 2015 presented 12 transitions between categories (change in forest cover density). The transitions in cover density varied dynamically, with almost all categories having losses in some zones and gains in others. When considering net changes between 1994 and 2015, the main winners were the Closed and Deforested categories, while the main losers were Non-Forest and Open (Table 4, Fig. 3).

**Table 4:** Area gains and losses by category and net change between 1994—2015 and 2015—2035

	1994 (ha)	2015 (ha)	Net Change 1994–2015 (ha)	2035 (ha)	Net Change 2015–2035 (ha)
Closed	428,072	487,057	58,985	512,803	25,746
Open	85,886	59,935	−25,951	51,198	−8,736
Deforested	55,554	43,849	−11,705	36,999	−6,850
Non-forest	165,761	144,433	−21,328	134,273	−10,160

The multinomial logistic regression showed that the likelihood ratio chi-square test was statistically significant, χ^2^(20) = 4,723, *p* < 0.0001, indicating our model containing the complete set of predictors fit the data significantly better than a null (or intercept-only) model. The resultant model was:$$ln\left(\frac{P(Y=Recovery)}{P(Y=Persistance}\right)=-.381+ {.000}_{elevation}+ {.000}_{population}- {.762}_{margination}- {.148}_{migration} + {.000}_{distance to roads}+ {.015}_{slope}+ {.000}_{distance to urban}+ {.684}_{protected areas}- {.221}_{PES}+ {.024}_{communal lands}$$

The general predictive performance of the model (the accuracy in classifying cases into the three categories on the dependent variable) showed that among the recovery areas, 69.9% were correctly predicted by the model to fall into this category. Among the areas of persistence, 55.1% were correctly classified to fall into this category. Finally, 0.0% of the disturbance areas were correctly predicted to fall into this category. The overall classification accuracy for the model was 45.7%.

Regarding the power of predictor variables to explain the changes, according to Cramer's V statistic (> 0.15), the variables associated with this process and to be included in the predictive model were Elevation, PES, and Distance to roads. These variables provide insight into the drivers of forest recovery and regional disturbance. Elevation limits agricultural expansion at higher altitudes, allowing forest regeneration where temperatures are cooler and terrain is less accessible. Road proximity is a critical factor in forest disturbance, as areas near roads experience more deforestation and degradation due to increased human activities. In contrast, remote areas are better protected. PES programs promote forest recovery by providing financial incentives to landowners for maintaining or restoring forest cover, leading to higher reforestation rates and afforestation. Communal lands, collectively managed by local communities, can support forest recovery through sustainable land management or contribute to degradation, depending on local governance and economic pressures.

The Chi-square test results to assess the potential influence of PES on forest recovery revealed a strong relationship between areas with payments for ecosystem services and those that underwent recovery (χ2 = 54.8, *p* < 0.0001).

Supposing that the same trends observed in the model calibration from 1994 to 2015 will be maintained for the 2015 to 2035 period, the predictive model showed an increment in the Closed category and decrements in the other three categories (Table 3, Fig. 3). For 2035, the forest will cover 77% of the study area, with the closed category covering 70%, representing an increment of 7% in the forest areas and 12% in the closed category relative to 1994.

**Fig. 4:**
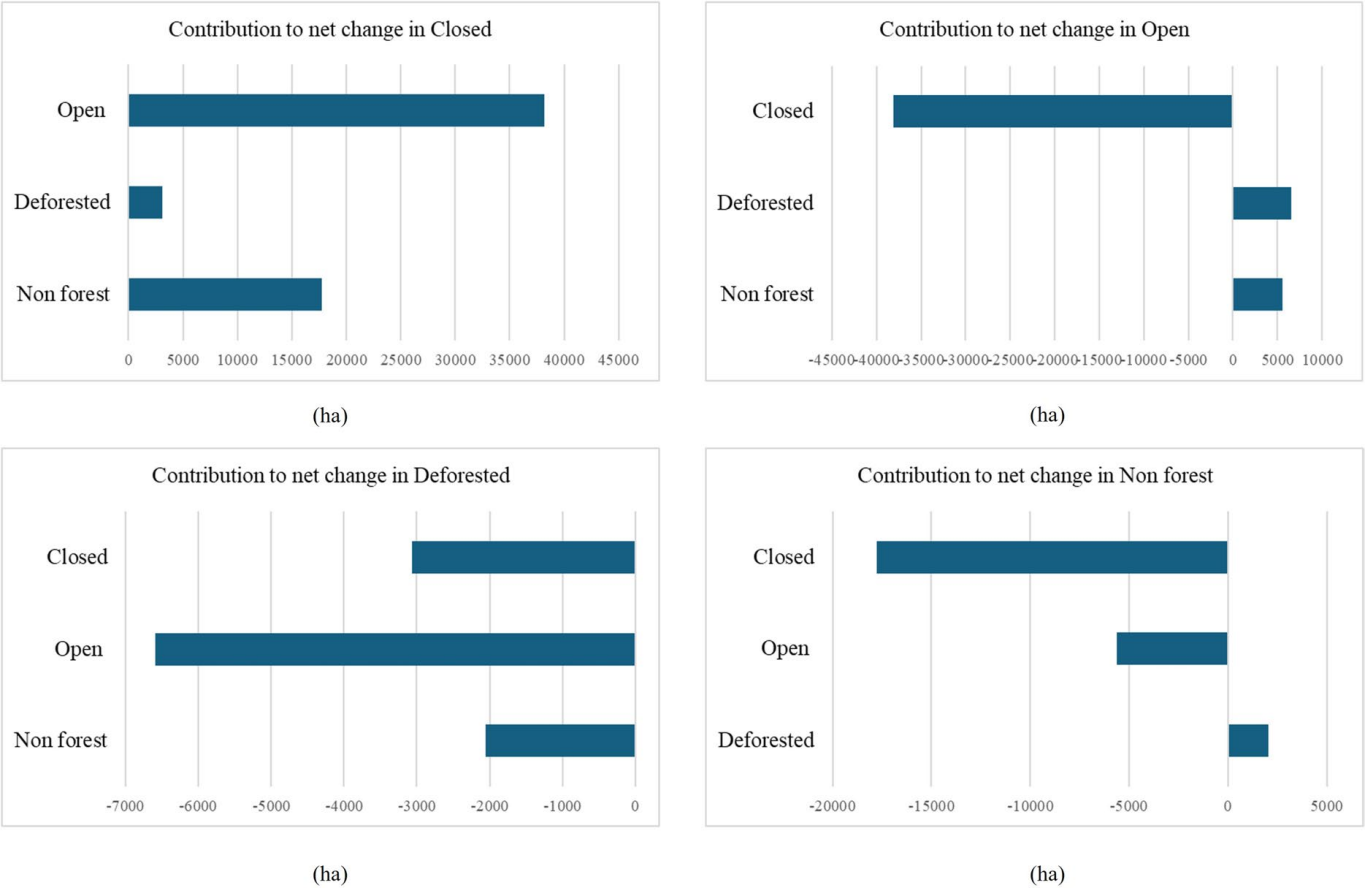
Net change for the cover categories 1994–2015

As mentioned above, the dominant process was recovery since all other categories contributed to the Closed category, mainly the Open category, followed by the Non-forest category. Deforested and Non-forest contributed to the Open category, while the latter two categories mostly had losses (Fig. 4).

## Discussion

This study highlights the persistence of land cover and the recovery in forest cover in Central Mexico from 1994 to 2015, with a net increase of 4.5%. These findings agree with Song et al. (2018), who reported that global tree cover increased from 1982 to 2016 with a net gain in the temperate forest. Densification of existing forests and establishment of new forested areas through afforestation have been crucial in forest recovery, aiming to restore degraded areas and enhance biodiversity conservation (Chazdon et al., 2020; Da Cruz et al., 2021). Forests have been recovering through natural processes and government reforestation programs. Abandoned agricultural and cattle lands have been afforested, and passive reforestation has occurred as cattle grazing and illegal logging have decreased in protected areas (Poorter et al., 2021). Besides the Mexican federal government's agricultural program, Procampo was expanded to include forestry so that idle or abandoned farmland could be reforested, with landowners receiving economic incentives and support for tree planting (DOF, 2019). These plantations are for conservation in communal lands and protected areas.

National and international reforestation efforts have been significantly boosted. The Bonn Challenge, launched in 2011, has secured commitments from 63 countries to restore millions of hectares of degraded and deforested land by 2030 (Verdone & Seidl, 2017). In Mexico, the national reforestation efforts have been funded by a combination of private organizations and federal government programs, including direct financial support for landowners who plant or maintain forested areas, as well as initiatives focused on forestry development and ecosystem restoration (Muñoz-Piña et al., 2008). For nearly five decades, the State of Mexico (included in this research) restricted private harvesting of forest resources through a conservation policy involving seasonal closures (de la Cruz-Hernández, 2016). In 1990, the State of Mexico established the public agency PROBOSQUE to protect, conserve, reforest, promote, and oversee the state's forest resources (Gaceta del Gobierno, 2023). This agency has since maintained annual reforestation programs.

Despite the positive trend in forest recovery, we also identified 4.5% of the study area with forest disturbance processes. This is not a surprise since the study area is surrounded by some of the country's main urban areas (including Mexico City), which contain around 37 million inhabitants, and because urban areas have been identified as a critical driver of forest loss and fragmentation (Zhou et al., 2017). The expansion of cities has been found to contribute significantly to forest degradation, leading to the depletion of surrounding forests (Von Thaden et al., 2021). This is often due to the need for agricultural land, firewood, and construction materials to meet the demands of the growing population (Hojas-Gascon et al., 2016).

The disturbances found in Central Mexico include deforestation, land-use change, and degradation. These processes have also been reported in several studies (Mesfin et al., 2020; Onilude & Vaz, 2020) and are associated with a decrease in ecosystem services (Wang et al., 2020). Other studies report a combination of positive and negative changes, as in Ecuador, where they are experiencing deforestation in some areas and forest regrowth in others (Haro-Carrión et al., 2018). The spatial distribution of the forest disturbances reveals a concentration outside areas with payment for environmental services and zones closer to roads, indicating that policy instruments and infrastructure development can significantly impact forest recovery (Ghazoul & Chazdon, 2017). These findings reinforced the Cramer V results, which highlighted the importance of Elevation, PES, and Distance to roads. The PES case is essential because several studies have evaluated its effects; in several cases, they have failed to achieve environmental and social benefits, but in some cases, they have promoted forest conservation and recovery (Denham, 2017; Ramirez‐Reyes, 2018). In two regions inside the study area (Sierras de las Cruces and Chichinautzín), the forest recovery was the result of the PES implementation starting in 2003 in 29.33% of the forest and the continuous programs of reforestation, fire control mainly in the State of Mexico, and surveillance by the local communities (López-García & Navarro Cerrillo, 2021). The results of those studies are consistent with this study where we found a strong association between PES application and forest recovery. On the contrary, roads have been reported as one of the main drivers of disturbance, as they influence the expansion of the agricultural frontier and give access to the extraction of natural resources, producing forest loss (Hernández-Flores et al., 2017; Lin et al., 2020; Motlaq et al., 2018; Nahar et al., 2023). The influence of elevation on land use change is complex and varies depending on the geographical context. It has been documented that lower altitude areas are the most likely to become urban (Megahed et al., 2015). In contrast, higher elevations tend to have cooler temperatures and more precipitation, making them more suitable for forestry or conservation than agriculture or human settlement. (Halofsky et al., 2017; Meyfroidt & Lambin, 2011).

Protected areas and communal lands are pivotal in shaping land use and land cover change dynamics. Protected areas often function as barriers against deforestation and land degradation by enforcing conservation regulations, limiting human activities, and promoting forest recovery (Burivalova et al., 2019). In this study, forest recovery within protected areas was notable, driven by natural processes and government-led reforestation efforts. Communal lands like ejidos play a complex role in land use and cover changes. Community involvement and PES programs can promote sustainable forest management, but weak governance can make these lands vulnerable to deforestation. The interaction between governance structures and policies is crucial in understanding the region's forest recovery and disturbance patterns, providing insights into how land tenure systems impact conservation (Hajjar et al., 2021).

Given the dynamic nature of land use change and forest cover, implementing robust monitoring systems and focused management strategies is essential in enhancing forest recovery efforts. Applying a detailed analysis of the forest cover through visual interpretation of high-resolution remote sensing data allows the tracking of changes in forest health, identifying the specific forest dynamic processes such as degradation, deforestation, densification, reforestation, and afforestation, assessing the balance between recovery and disturbance, and establishing forest restoration and management programs.

However, visual interpretation, although more accurate than other methods, is time-consuming, and finding historical data of the necessary quality is challenging. Another limitation is the temporal scope, as the research focuses on changes between two-time points. This may miss intermediate fluctuations that could provide a more nuanced understanding of the recovery and disturbance dynamics. Additional time points could have offered a clearer picture of the trends and identified critical periods of change. Finally, the analysis did not consider the influence of human activities, such as illegal logging, small-scale agriculture, and urban expansion, which can have significant localized impacts on forest cover.

The results of this study highlight the urgent need for specific policies and management strategies to maintain and improve the forests in Central Mexico. These strategies must consider that degradation leads to declining ecosystem function due to unsustainable land use practices, such as overgrazing or logging, which reduce biodiversity, deplete soil nutrients, and increase erosion. Deforestation, driven by agricultural expansion, urbanization, or illegal logging, causes habitat loss, disrupts water cycles, and contributes to global warming by releasing stored carbon (IPBES, 2018). On the other hand, afforestation, the practice of planting trees in areas that were not historically forested, can be harmful if not carefully planned. This practice can disrupt local ecosystems by introducing non-native tree species, threatening biodiversity, and displacing native flora and fauna. Additionally, large-scale afforestation projects can interfere with hydrological processes, such as water availability and groundwater recharge, by increasing water consumption through tree transpiration (Xiao et al., 2020). Therefore, afforestation projects must be ecologically appropriate and meticulously managed to mitigate unintended negative consequences.

The research identified some of the factors that influence forest recovery, providing information for future conservation efforts. Among these factors, Payment for Environmental Services programs have proven effective in preventing forest degradation (Denham, 2017; Ramirez‐Reyes, 2018). Expanding their funding and reach, especially in areas where forest recovery is slower, can encourage greater stakeholder participation and amplify the impact of conservation efforts. In addition, the study points to the proximity of roads as a major driver of forest disturbance. Road infrastructure construction often results in habitat fragmentation and increased accessibility to forests, making them more vulnerable to exploitation (Hernández-Flores et al., 2017; Lin et al., 2020; Motlaq et al., 2018; Nahar et al., 2023). To mitigate these adverse effects, road construction must be carefully planned, avoiding constructing new roads in ecologically sensitive areas and implementing measures to reduce the impact of existing infrastructure, such as establishing buffer zones or encouraging reforestation along roads.

The study reveals both positive trends in forest recovery and disturbance. This underscores the need to monitor forest cover changes continuously. Understanding long-term trends is crucial for assessing the effectiveness of management strategies and policies promoting forest conservation (Mehmood et al., 2024). Using predictive models to forecast future trends in forest cover can alert policymakers to potential changes, allowing them to anticipate and plan accordingly. Using predictive models, conservation efforts can become more proactive, helping sustain forest recovery in the long term. The study's projected scenario is based on the continued observed land use and land cover trends from 1994 to 2015. It assumes that forest recovery, driven by densification, afforestation, and reforestation, will persist, leading to a 7% increase in total forest cover by 2035. These results should be analyzed, considering that while the Land Change Modeler is a powerful tool for analyzing land use and land-cover change, has inherent limitations. Its use of historical data and machine learning can result in biased predictions that fail to account for potential future changes. Additionally, spatial allocation of changes can be influenced by stochastic variability and neighborhood effects, potentially misallocating changes to areas with lower transition potential (Noszczyk, 2018) (See the Supplementary Information for transition potential classes and predicted change areas). Key factors like elevation, proximity to roads, and payment for environmental services programs are expected to keep influencing these dynamics, with PES areas promoting recovery and roads contributing to disturbances. Protected areas are predicted to maintain their role in conserving forest cover, further reinforcing positive trends.

Although this study focuses on forest recovery in Central Mexico, its findings have broader implications for global forest management and conservation. The dynamics of forest densification, afforestation, and the role of socioeconomic drivers such as migration and payment for environmental services (PES) programs reflect common challenges in many regions worldwide. Using high-resolution spatial data and predictive modeling, the study offers a framework that can be adapted to other geographic contexts facing similar deforestation, land degradation, and recovery issues. Furthermore, the lessons learned from this localized case can inform international efforts under initiatives like the Bonn Challenge and global climate action, where the balance between natural regeneration and active reforestation is critical to restoring ecosystems, mitigating climate change, and conserving biodiversity globally.

## Conclusion

This study examined land cover changes over 21 years in the mountainous regions of Central Mexico, exploring the factors driving forest recovery. Various disturbance and recovery processes, including degradation, deforestation, densification, reforestation, and afforestation, were identified using high-resolution remote sensing data and a detailed land cover density classification. Findings showed the progress in forest recovery between 1994 and 2015, mainly through densification and afforestation. The analysis identified key factors influencing forest recovery, such as elevation, distance from roads, and participation in payment for environmental services (PES) programs. These findings suggest that strategic conservation efforts focused on high-elevation areas and regions less accessible by road can further enhance forest recovery. Expanding PES programs could provide continued incentives for landholders to engage in forest conservation. The predictive modeling to 2035 suggests that forest areas will continue to grow, with the closed forest category expected to dominate the landscape. While our study has revealed positive trends in forest recovery, degradation, and deforestation, challenges persist. Addressing these challenges will require concerted efforts from policymakers, stakeholders, and local communities to strengthen conservation policies, monitoring efforts, and sustainable land management mechanisms. The detailed land cover density classification system could enhance the accuracy of forest monitoring efforts and can be adopted in other regions to support improved forest management and conservation strategies. Further research should focus on enhancing data's spatial and temporal resolution, explicitly incorporating human decision-making's influence on land use modeling, and conducting interdisciplinary collaborations to deepen our understanding of forest dynamics.

## Supplementary Information

Below is the link to the electronic supplementary material.

Supplementary file1 (DOCX 1137 KB)

## Data Availability

Data will be made available on request.
