# Supplementary material for: A long-term analysis, modeling and drivers of forest recovery in Central Mexico
Source: Environ Monit Assess. 2024 Dec 21;197(1):87. doi: 10.1007/s10661-024-13584-0 (PMC11663157; doi:10.1007/s10661-024-13584-0)
Supplement: Supplementary file 1 — Supplementary file1 (DOCX 1137 KB) [file 10661_2024_13584_MOESM1_ESM.docx]

**Environmental Monitoring and Assessment**

**A Long-term Analysis, Modeling and Drivers of Forest Recovery in Central Mexico**

José López-García^1^, Gustavo Manuel Cruz-Bello*^2^, Lilia de Lourdes Manzo-Delgado^1^

^1^ Instituto de Geografía, Universidad Nacional Autónoma de México, Ciudad Universitaria, C.P. 04510 Coyoacán, Mexico City, Mexico

^2^ Departamento de Ciencias Sociales, Universidad Autónoma Metropolitana, Unidad Cuajimalpa. Avenida Vasco de Quiroga 4871, Col. Santa Fe, C.P. 05348, Mexico City, Mexico.

^*^Corresponding author: [gcruz@cua.uam.mx](mailto:gcruz@cua.uam.mx) (GMCB)

**Examples of three densities**


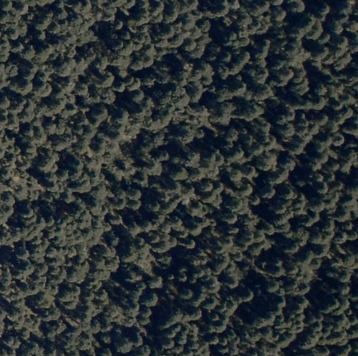


Closed cover (>50% density)


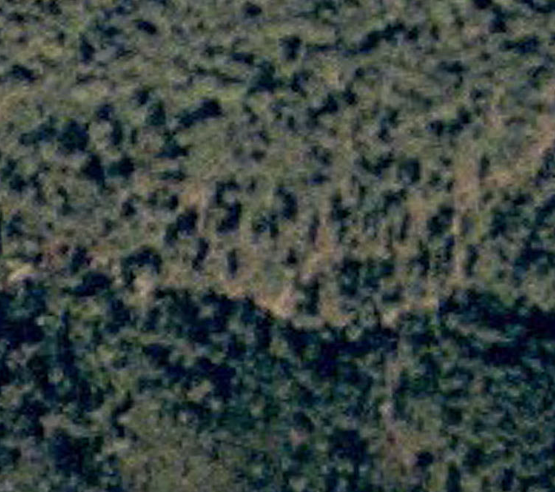


Open cover (<50% density)


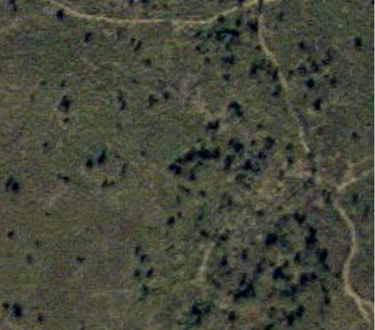


Deforested (<10% density)

**Predicted Change by Transition Potential
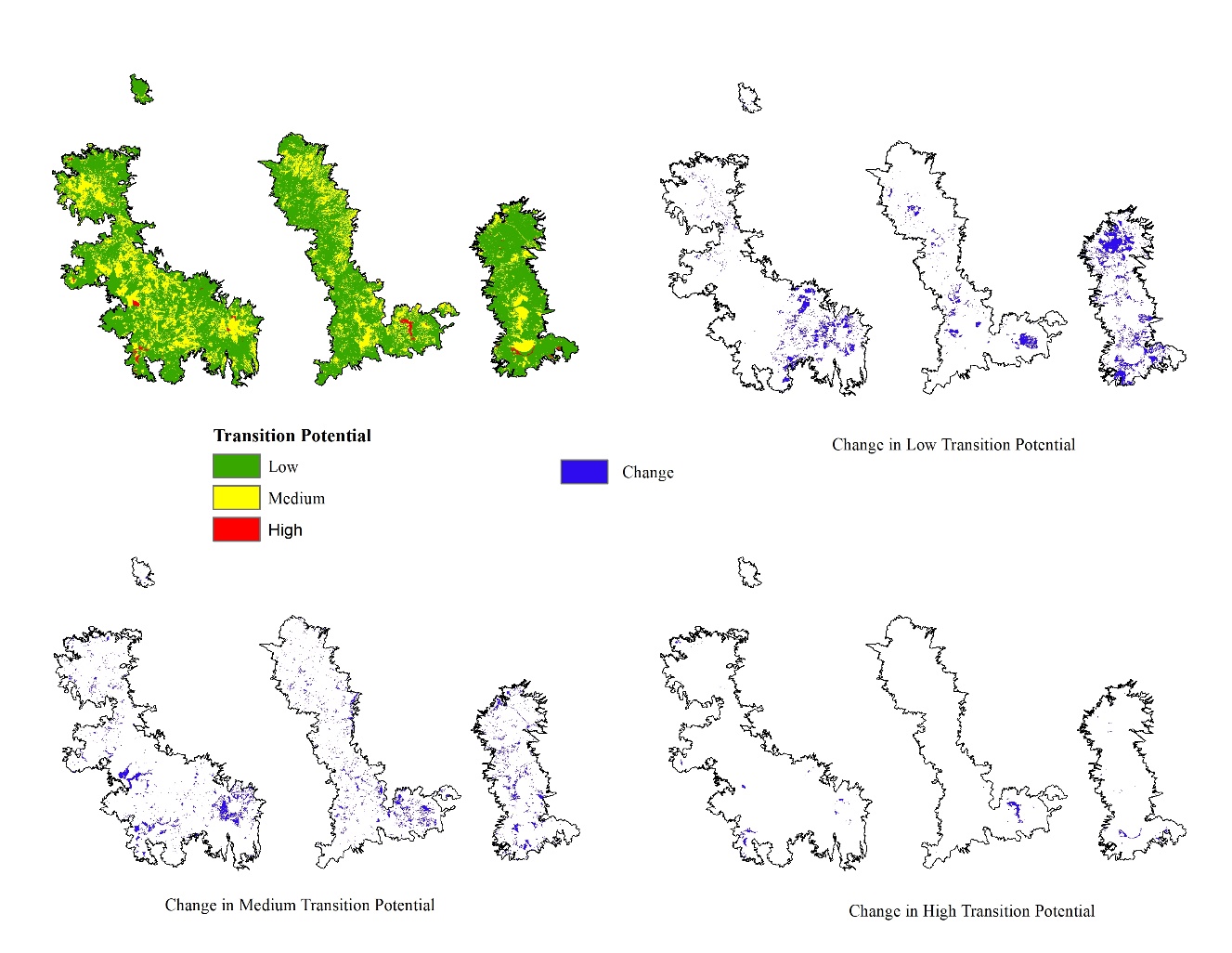
**
